# Fourth revolution in psychiatry – Addressing comorbidity with chronic physical disorders

**DOI:** 10.4103/0019-5545.70973

**Published:** 2010

**Authors:** Shiv Gautam

**Affiliations:** Department of Psychiatry and Superintendent, Psychiatric Center, Jaipur, India

**Keywords:** Fourth revolution, Psychiatric comorbidity, chronic physical disorder

## Abstract

The moral treatment of mental patients, Electro Convulsive therapy (ECT), and Psychotropic medications constitute the first, second, and third revolution in psychiatry, respectively. Addressing comorbidities of mental illnesses with chronic physical illnesses will be the fourth revolution in psychiatry. Mind and body are inseparable; there is a bidirectional relationship between psyche and soma, each influencing the other. Plausible biochemical explanations are appearing at an astonishing rate. Psychiatric comorbidity with many chronic physical disorders has remained neglected. Such comorbidity with cardiac, respiratory, Gastrointestinal, endocrinal, and neurological disorders, trauma, and other conditions like HIV and so on, needs to be addressed too. Evidence base of prevalence and causal relationship of psychiatric comorbidities in these disorders has been highlighted and strategies to meet the challenge of comorbidity have been indicated.

## INTRODUCTION

At the outset, I would like to express my gratitude for considering me to deliver this prestigious oration instituted by the Indian Psychiatric Society in memory of late Professor DLN Murthy Rao, a great teacher, scholar, clinician. and healer. I have been a student of Professor DLN Murthy Rao and have been trained in the institution where he worked. I have felt his aura in the teachings of my teachers and am delighted to be here before you.

The first revolution in psychiatry is generally acknowledged to be the unchaining and moral treatment offered to mental patients by Pinel in the year, 1793.[1] The second revolution was heralded by the invention of electroconvulsive therapy (ECT) in the year 1935, by Cerletti and Bini.[2] It was the first effective and easily feasible treatment option for a variety of mental illnesses. Another leap for psychiatry was the introduction of psychotropic agents, chlorpromazine to be particular, in the year 1952, and the later discovery of a series of antidepressants, anti-anxiety, anti-psychotic, and other neuroleptic drugs. It changed the face of psychiatry forever and allowed domiciliary treatment.[3] This is generally regarded as the third revolution of psychiatry and combined with the treatment of the mentally ill outside the four walls of the mental hospital has revolutionized the outcome of mental illnesses.

Addressing comorbidities of mental illnesses with chronic physical illnesses will be the fourth revolution in psychiatry. Mind and body are inseparable and there is a bidirectional relationship between psyche and soma, each influencing the other. Psychological factors must be taken into account when considering all disease states. Physical diseases have a large overlap with mental disorders. All physical illnesses and their management cause a psychological reaction. This may or may not reach morbid levels, similarly mental illnesses and stress predispose to a large variety of physical illnesses. A bidirectional relationship has been established and the evidence grows by the day. Plausible biochemical explanations are appearing at an astonishing rate. We are all aware of the neurochemical response, immune response, and endocrine response to stress.[4]

The catabolism of tryptophan is stimulated under the influence of stress, hormones, and inflammation, by the induction of the enzyme tryptophan pyrrolase. On account of the reduction in blood levels of tryptophan under these circumstances, the formation of cerebral serotonin is decreased. Depression is associated with many chronic disorders and aging: in each case depressed individuals have the worse outcome. In all these conditions there is now evidence of impaired phospholipid metabolism and impaired fatty acid-related signal transduction processes. This may be a primary cause of depression in chronic illnesses.[5]

Addressing psychiatric comorbidities with chronic physical disorders shall be the fourth revolution in psychiatry. Let us look at the comorbidities with various chronic physical disorders and strategies of treating them.

## COMMON PHYSICAL DISORDERS WITH PSYCHIATRIC COMORBIDITY

**Cardiovascular disorders**
  Coronary artery disease
  Arrythmia
  Hypertension
  Congestive heart failure
  Mitral valve prolapse
**Respiratory system**
  Asthma
  COPD
  Pneumonia
  Embolism
  Pneumothorax
**Gastrointestinal**
  Peptic ulcer
  IBS
  Celiac disease
**Endocrine**
  Thyroid disorders
  Diabetes
  Cushing’s syndrome
**Neurological**
  Epilepsy
  Parkinsonism
  Stroke
  Alzheimer’s disease
  Encephalopathy
**Miscellaneous**
  Accidents
  HIV
  Cancer
  Skin disorders

## PREVALENCE OF DEPRESSION IN VARIOUS PHYSICAL DISORDERS

It has been reported that prevalence of depression associated with various chronic physical disorders is comparatively higher than in the general population.[6]

**Table:** 

General population	17%
Post-partum	51%
Diabetes	27%
HIV	45%
Coronary artery disease	45%
Stroke	80%

### Cardiovascular disorders

Cardiovascular disorders are one of the leading causes of death. Depression, anxiety, type A behavior, hostility, and the like, have all been evaluated as risk factors for cardiac disease. A bidirectional relationship has been noted. Sixteen to twenty-three percent prevalence of depression is noted in patients with Coronary Artery Disease. Acute emotions can cause autonomic arousal and thereby precipitate arrhythmia in the predisposed.[7] Hypertension and its relationship with certain personality types has been widely reported.[8] Acutely stressful situations are known etiological factors of vasovagal syncope.

- One plausible contributing mechanism is the tendency of those with psychiatric disorders to ruminate on stressful events. This phenomenon, sometimes called perseverative cognition, can extend to the psychological and physiological effects of stress, which could contribute to cardiovascular disease etiology[9] Certain genes (5-HTTLPR and STin2 VNTR, but not the rs25531), responsible for polymorphisms of SERT (serotonin reuptake transporter), are associated with post stroke depression (PSD) in stroke survivors and patients with MI (myocardial infarction). This gives further evidence for a role of SERT polymorphisms in mediating resilience to biopsychosocial stress.[10] In a sample of outpatients with CAD (coronary heart disease), the association between depressive symptoms and adverse cardiovascular events was largely explained by behavioral factors, particularly physical inactivity.[10]

After adjustments for gender, age, ethnicity, education, and employment status, sympathetic arousal and early-morning insomnia were significantly associated with cardiac disease.[11]

In the setting of cardiovascular rehabilitation, approximately 45% met the criteria for at least one anxiety disorder, and 20% met the criteria for either major depressive disorder or dysthymic disorder at the time of evaluation or in their lifetime. Across all participants, 26% met yjr criteria for at least two PD (personality disorders).[12]

In CHF (congestive heart failure) patients as a whole, 20% of the patients met the DSM-IV criteria for a cu r rent major depressive episode, 16% for a minor depressive episode, and 51% scored above the cutoff for depression on the Beck Depression Inventory (>10).[13]

Vijayvergia and Vyas (1987), reported that essential hypertensives perceived more number of stressful life events and were unable to express themselves. Certain personality factors associated with hypertension were reported to be, being reserved, detached, cool, emotionally less stable, with low frustration tolerance, and so on.[14] Patients undergoing open heart surgery had pre- and postoperative psychiatric disturbances. Anxiety, depression, and delirium were the common diagnosis. (Shekhawat and Gautam (1994))[15] Sharmaand Gautam (1997), reported that 80% of Non-Cardiac Chest Pain patients identified psychological factors as the precipitating factor; 67.5% of the patients had psychiatric morbidity. (Studies carried out at PCJ)[16]

### Respiratory disorders

Disturbances of breathing can perturb psychic calm as in the terror of any asthmatic patient. Likewise, psychological distress may become evident by disrupted breathing as seen in depressed and anxious patients.

At least half of the children with anxiety disorders had a comorbid physical illness. Allergies and asthma were the most common comorbid physical illnesses.[17]

When comorbid with COPD (chronic obstructive pulmonary disease), mental health symptoms of depression and anxiety are some of the most salient factors associated with quality-of-life outcomes. A possible causal effect of depression on COPD exacerbations and hospitalizations has been suggested.[18] Chronic bronchitis is strongly associated with depression and anxiety.[19] As depression and/or anxiety may not only interfere with an attempt to stop smoking, but also contribute significantly to experiencing low quality of life, it is important to consider these disorders.[20]

Trials of nortriptyline, buspirone, and sertraline have been found to reduce symptoms of anxiety in the Patients of COPD. Similarly, cognitive–behavioral programs that focus on relaxation and changes in thinking also produce declines in anxious symptoms.[21] Finally, multicomponent pulmonary rehabilitation programs can also result in reductions in anxious symptoms.[22]

Tyagi and Vyas (1989), reported that 65% of asthmatics suffered from psychiatric morbidity, chief among them being anxiety and depression. (Study carried out at PCJ)[23]

### Gastrointestinal disorders

Gastrointestinal disorders are very prevalent and a large proportion of these disorders are functional in nature. Psychological and psychiatric factors commonly influence the onset, severity, and outcome of many gastrointestinal disorders. IBS (Irritable bowel syndrome) and psychiatric illness have high rates of bi-directional comorbidity;[24] 35.1% of the patients with OCD (Obsessive–compulsive disorder) satisfied criteria for IBS. SSRIs (Selective serotonin reuptake inhibitors) could potentially worsen such symptoms and lead to non-adherence.[25] The prevalence of IBS and other functional gastrointestinal disorders with panic disorder were substantially higher[26] Improved depression was associated with improved role functioning.[27] Cognitive-behavioral therapy (CBT) has received increased attention in light of a recent shift in the conceptualization of IBS as a disorder of brain–gut function.[28] One-third of the 30 patients with IBS suffered from psychiatric comorbidity and perceived a greater number of stressful life events.(Arun and Vyas(1989), (study conducted at PCJ)[29]

Any childhood abuse was associated with a significantly increased odds ratio for recurring stomach problems, and frequent childhood abuse was associated with a significantly increased likelihood of recurring stomach problems and ulcer.[30] Generalized anxiety disorder (GAD) was associated with a significantly increased risk of self-reported PUD (Peptic Ulcer Disease). Peptic ulcer patients perceived more number of stressful life events and had higher alexithymia scores.(Banerjee and Vyas (1988), study conducted at PCJ)[31]

A high prevalence of depressive symptoms, hypothetically related to serotonergic dysfunction, have been reported among adults with celiac disease[32] Adolescent celiac disease patients with depression have significantly lower pre-diet tryptophan/competing amino-acid (CAA) ratios and free tryptophan concentrations, and significantly higher biopsy morning prolactin levels compared to those without depression[33]

### Diabetes

Psychiatric comorbidity with diabetes is common and impacts its course and outcome. Up to 30% of such patients are depressed, and anxiety disorders are also very common. A bidirectional relationship is apparent. People with depression have poorer glycemic control and depressed people are prone to develop diabetes.[34] Depressive symptoms were associated with increased risk of MCI (Mild Cognitive Impairment), and this association was independent of the underlying vascular disease.[35] People with diabetes, smoking, and obesity were associated with a greater likelihood of meeting the criteria for major and minor depression.[36] Among patients with diabetes, both minor and major depression are strongly associated with increased mortality.[37] Depressive symptom severity is associated with poorer diet and medication regimen adherence, functional impairment, and higher healthcare costs in primary care diabetic patients.[38] The occurrence of eating disorders was increased compared to the rates observed in the general population, with the predominance of binge eating disorder. Dubey and Solanki (2004), studied cognitive impairment and depression in diabetes and found that 48% of the diabetics showed cognitive impairment and 36% of the patients were suffering from depression.[39] Sushil and Vyas (1990), reported that 74% of the diabetics suffered from psychiatric comorbidity; 44%–depression, 10% – mixed anxiety and depression, 14% – anxiety neurosis, 2% – phobia, and 4% – sexual problems. (Studies carried out at PCJ)[40]

### Thyroid

Hyperthyroidism may present with psychiatric symptoms like anxiety, irritability, lability, fatigue, restlessness, and so forth. Hypothyroidism can cause depression, cognitive impairment, and rapid cycling mood disorder. Subclinical hypothyroidism is also an important cause of depression. The relationship between basal thyroid hormone levels and acute antidepressant response has been studied. Time to recurrence of major depression was inversely related to T3 levels and not to T4 levels.[41] Erectile Dysfunction (ED) is extremely common in males with dysthyroidism. Treatment of the latter restores erectile function.[42] A significant association of subclinical hypothyroidism with psychiatric disorders and an increased frequency of subsyndromic depression and anxiety symptoms is reported.[43] Jain and Gautam (1988), reported that 58.33% of patients with thyroid dysfunction had psychiatric illnesses (n=60). (Study carried out at PCJ)[44]

### Central nervous system

Headaches are the most common neurological complaint and a major cause of absenteeism. Most headaches have an emotional basis and even headaches with neurological basis have significant psychiatric comorbidity. All headache types are reported to be more prevalent in depressed patients; the strongest association being between depression and migraine with aura[45] Elevated one-year prevalence rates for a wide range of psychiatric disorders (Anxiety spectrum disorder, depression, bipolar affective disorder) in people with migraine has been reported[46] Dysregulation of serotonergic neurotransmission has been postulated to have a key role in the pathogenesis of both major depression and migraine.

### Epilepsy

At least a two-fold, overall increase in psychiatric morbidity in patients with epilepsy has been noted. In the pre-ictal state, prodromal states and mood disturbances are commonly seen. During the ictal state of complex partial seizures, the disturbances often seen are affective disturbances, hallucinations, experiential phenomenon, and automatisms. Impaired consciousness, delirium, psychosis, and Todd’s paresis are usually seen in the post-ictal phase. During the inter-ictal stage, the commonly seen disturbances include cognitive, psychoses, sexual behavior, depression, suicide, crime, antisocial behavior, and personality change.

Latest evidence shows a significant association between the prevalence of depressive symptoms and non-lesional focal epilepsy[47] Simple causal links between epilepsies and psychoses appear increasingly tenuous, despite indications that some psychotic symptoms and some localized structural changes are linked

In a study of 204 patients, Guerje *et al*. found that 37% emerged as psychiatric cases, almost a third of these being cases of psychosis. Patients with partial seizure of the temporal lobe origin were the most psychiatrically impaired. Self-poisoning is a common complication of epilepsy. Epilpeptics have been found to have less alcohol excess, but significantly more psychopathy[48] Purohit and Satija (1984), reported that psychiatric manifestations were present in 35% of the cases of chronic epilepsy and they were more common in temporal lobe epilepsy. (Study conducted at PCJ)[49]

### Stroke

Overall 31.8–35.5% of the stroke patients have depression. They are likely to be underestimated due to under reporting of unusual mood, difficulties in assessment of depression in neurologically impaired individuals, and variability in the methods used to assess and define depression.[50]

### Alzheimer’s disease

At the initial evaluation, 19% of the Alzheimer‘s’disease patients had major depression and 34% had dysthymia, after a mean follow-up of 16 months; 58% of the patients with major depression at the initial evaluation were still depressed, whereas, only 28% of the patients with initial dysthymia and 21% of the non-depressed patients were depressed at follow-up. All three groups showed similar declines in cognitive status and activities of daily living.[51]

### Parkinson’s disease

The association of depression with Parkinson’s disease is well-established, with a prevalence of 40%. Psychotic symptoms occur at some stage in 20% of the patients and excessive somnolence, day-time sleepiness, and sleep attacks are also common. Research has suggested that high levels of depression and anxiety observed in Parkinson’s disease are a primary consequence of its pathophysiology. However, people with a specific metacognitive style had an increased vulnerability to distress over and above the previously identified disease factors[52]

### Head injury

Psychiatric disability has been found to correlate to a statistically significant extent with the depth and quantity of brain damage. The duration of post-traumatic amnesia and the incidence of post-traumatic epilepsy show significant correlation. Similarly, the development of epilepsy, especially if within one year of injury, is associated with increased psychiatric disability. Left hemisphere lesions and temporal lobe wounds are more closely associated with psychiatric disability.[53]

### Other accidents

Bhojak and Gehlot, (1982) reported that 93.33% of limb amputees reported phantom limb phenomenon, 63.33% of the patients were depressed, and 46.66% were suicidal (n=30)[54] Mordia and Gautam (2000), found that there is a significant psychiatric morbidity post- burn (62.86%), which includes depression (21.43%), adjustment disorder (21.43%), PTSD (15.71%), and post-schizophrenic depression (4.26%) (Studies conducted at PCJ)[55]

### AIDS

Psychiatric disorders in populations of people with HIV exceed the general population estimates significantly. Rates of depression range from 20 to 37% in people with HIV. Cognitive impairment increases as the immune system worsens and HIV progresses. Furthermore, the prevalence of minor HIV-associated cognitive impairment is rising among patients on HAART (highly active anti-viral therapy) as a result of increased survival time.[56] Youths with major mental disorders had a high prevalence of most HIV-AIDS risk behaviors. Comorbid substance use disorders substantially increased the risk.[57] HIV-associated neurocognitive disorders (HAND) are common among HIV patients, and HIV-associated dementia (HAD) is a serious condition. The introduction of HAART has resulted in a significant decrease in morbidity and mortality in HIV-infected patients. HAART has also decreased the incidence of HAD, but does not give complete protection. The utility of psychotropic medications in HIV patients has not been studied sufficiently. Mandal and Bhojak (2005), reported that a prevalence of depression and anxiety in HIV-positive patients was 26 and 16%, respectively. (Study carried out at PCJ)[58]

### Cancer

Comorbidity in cancer is due to the response to the diagnosis — anxiety, shock, denial, depression, adjustment disorder, suicide. Major depression occurs in 10–20% of the patients in the course of the disease. Progression and recurrence are often associated with increased psychiatric disturbance. Para-neoplasia metastasis and style of coping predicts survival in lung cancer.[59] Cancer patients with depression have markedly higher plasma concentrations of IL-6 than healthy comparison subjects and cancer patients without depression.[60] The treatment of penile cancer results in negative effects on the well-being in up to 40%, with psychiatric symptoms in approximately 50%. Up to two-thirds of the patients report a reduction in sexual function[61] Nijhawan and Gehlot (1982), reported that 78% of cancer patients suffered from psychiatric symptomatology and more than two-thirds of these patients suffered from depression.[62] Gautam and Nijhawan (1983), found that there was no significant difference in psychiatric morbidity between cancer patients and patients with chronic lung disease. (Studies conducted at PCJ).[63]

### Dermatology

Psychocutaneous disorders encompass a wide variety of dermatological diseases that may be affected by the presence of psychiatric symptoms or stress and psychiatric illness in which the skin is the target of disordered thinking, behavior or perceptions. Atopic dermatitis, psoariasis, psychogenic excoriation, pruritis ani, pruritis vulvae, hyperhidrosis, urticaria, leprosy, and so on, have a large psychiatric overlap. Meel and Gautam (1988), found that patients of dermatitis perceived more stressful life events, had schizothymic traits, and had lower scholastic mental capacity and lower ego strength. They scored high on the Toronto-Alexithymia scale. (Study carried out at PCJ).[64]

### Strategies to address psychiatric comorbidities with chronic physical disorders:

1. Training of general practitioners (GPs) and physicians in the identification and management of psychiatric comorbidities in rural and urban areas.
2. Sensitization of physicians working in different specialities in secondary and tertiary care hospitals.
3. Presentation of success stories of outcomes in medical and other outpatient departments.
4. Creating public awareness about psychiatric comorbidities with chronic physical disorders.

In a large number of patients suffering from psychiatric comorbidity with chronic physical disorders, the management of emotional problems remain neglected. Therefore, if a dent can be made in the management of the psychiatric comorbidities with chronic physical disorders, it would not only give a due status to the speciality of psychiatry, but would also change the quality of life of millions of patients.
